# Protocol: New approaches to managing the social deficits of Turner Syndrome using the PEERS program

**DOI:** 10.12688/f1000research.15489.2

**Published:** 2019-03-18

**Authors:** Jeanne Wolstencroft, William Mandy, David Skuse

**Affiliations:** 1 Great Ormond Street Institute of Child Health, University College London, London, WC1N 1EH, UK; 2 Clinical, Education and Health Psychology, University College London, London, WC1E 6BT, UK

**Keywords:** social skills training, social skills, peers, turner syndrome, sex chromosome aneuploidy

## Abstract

Turner Syndrome (TS) is a sex chromosome aneuploidy (45,X) associated with social skill difficulties. Recent clinical care guidelines recommend that the Program for the Education and Enrichment of Relational Skills (PEERS) social skills intervention programme be trialled in this population. PEERS has been successfully used in adolescents with autism spectrum conditions without intellectual disabilities. The PEERS program will be piloted with adolescents and young women with TS aged 16-20 using an uncontrolled study trial with a multiple-case series design. The program will be delivered face to face and online. The assessment battery is designed to measure social skills comprehensively from diverse informants (parent, teacher young person). It includes measures of social performance, social knowledge and social cognition. Parents and young people taking part in the intervention will also feedback on the acceptability and feasibility of the pilot. The outcomes of this small scale pilot (n=6-10) will be used to adapt the programme based on feedback and estimate the sample for a future randomised controlled trial.

## Introduction

Turner Syndrome (45,X; TS) is one of the most common sex chromosome aneuploidies, with an incidence of 1 in 2500 female births ^1^. TS is associated with a variety of morbidities affecting nearly every bodily system, including skeletal abnormalities such as short stature, dysmorphic features, hearing difficulties, infertility, cardiac abnormalities, diabetes and thyroid problems. These difficulties have been well characterized in the literature (see Gravholt *et al.* 2017 ^2^ for the most recent review) and require clinical monitoring across the lifespan.

TS females have social difficulties throughout childhood, but these become more apparent in adolescence when socialisation becomes more complex ^3^. Social deficits are exemplified by difficulties integrating within social groups, with poor deciphering and processing of social cues ^3^. Previous research has shown TS is associated with specific deficits in social cognitive competence, especially forming and maintaining peer relationships ^4^.

Some of the social deficits observed in TS are reminiscent of difficulties associated with Autism Spectrum Disorders (ASD). Psychosocial evaluations of young women with TS have found an association with ASD ^5– 9^, anxiety disorders, depression and low self-esteem ^10– 13^.

Social skills deficits are known to have a significant impact on academic, adaptive and psychological functioning ^14– 17^, and are likely to have a substantial impact on the wellbeing of girls and women with TS across the lifespan ^3^. At present, psychosocial intervention research with young women with TS is scarce; only one intervention targeting self-esteem in adults aged 18–30 has been documented in the literature ^10^. The latest TS Clinical Care Guidelines recommend that a social skills training intervention should be trialled in this population ^2^. They suggest using the Program for the Education and Enrichment of Relational Skills (PEERS) developed for children with ASD ^18^. There is good evidence for the efficacy of PEERS when delivered with children and young adults with ASD without intellectual disabilities ^19– 24^. This pilot project will be the first to examine the feasibility and acceptability of the PEERS Protocol in adolescents with TS.

## Protocol

### Objectives and hypothesis

The main objectives of the study are:

1) To pilot the PEERS intervention in adolescents with TS;
2) Assess its feasibility and acceptability to families.

We hypothesise social skills training will improve social competence with peers and may produce secondary improvements in social cognition, self-esteem and anxiety (social and generalised).

### Study design

We will be employing an uncontrolled trial design. To maximise the clinical reliability of the trial we will use a systematic multiple-case series design with case tracking. We aim to recruit participants with a similar degree of social impairment, and intellectual ability.

### Sample size

A sample size of 6–10 girls and their parents will be invited to take part in the study - this is the group size recommended by the PEERS intervention manual. At present the effect size for this intervention in girls with TS is unknown. This pilot will serve as the basis to estimate the intervention’s effect size and sample size for a future randomised control trial.

### Study centres/Recruitment

Participants will be recruited from the *Social Skills and Relationships in Turner Syndrome Study* (SOAR), which recruits children and young women with TS from the Turner Syndrome Support Society, the NHS Great Ormond Street Hospital and the NHS University College London Hospitals. 

The SOAR study is conducting online mental health and social cognition questionnaires with 200 girls and young women with TS and their parents. A subset of families from this large cohort that meet the trial’s inclusion criteria will be invited to take part in the intervention study.

### Participant inclusion and exclusion criteria

Inclusion criteria for the intervention include: 1, a confirmed diagnosis of TS (monosomy, variant, mosaic etc.); 2, age 16–20 years; 3, significant social skills difficulties as screened for in the SOAR online questionnaires (see screening assessment measures section for details) and clinical judgement; 4, motivation to take part.

The exclusion criteria for the intervention include: 1, profound hearing or vision impairments (eg. complete deafness or blindness); 2, intellectual disability (VIQ<70); 3, concurrent participation in other psychological treatment.

### Intervention

The UCLA PEERS for Adolescents is a manualized treatment program that consists of 14 90 min sessions ^18^. The program runs two concurrent groups, one for the adolescents and one for parents. At the end of each session the two groups are reunited for review and questions. Between sessions the adolescent group are given homework tasks, which they are to complete with the help of their parent who is trained to support them as their social coach. Parents are provided with concise handouts for each session, which include an overview of the lesson material and the homework.

The adolescent group sessions are structured to provide didactic instruction as well as social skill rehearsal. The parent sessions mirror the adolescent sessions and provide a space for the parents to problem-solve any difficulties they may have encountered the previous week. The didactic lessons provide instruction on (a) conversational skills; (b) electronic forms of communication; (c) developing friendship networks and finding sources of friends; (d) appropriate use of humour; (e) peer entry strategies; (f) peer exit strategies; (g) organizing get-togethers with friends; (h) handling teasing and embarrassing feedback; and (i) resolving arguments with friends ^18^.

The adolescents and parents will attend separate concurrent sessions led by a certified PEERS Instructor. Three face to face sessions will take place in London at the start, middle and end of the program. All other sessions will be conducted online using a virtual meeting room. The face to face sessions will deliver two PEERS lessons, whereas the weekly online sessions will deliver one lesson. Research assistants (graduate or undergraduate psychology students) will monitor treatment fidelity, assist with role-playing demonstrations, and provide social coaching with performance feedback during behavioural rehearsal exercises. All research assistants will be trained and supervised throughout the intervention.

### Assessments

Participants will complete assessments at different time points throughout the study. The study will last 9 months in total, including a 3 month baseline, 2 months of intervention and a 3 month follow-up period. The screening measures will be delivered at T=0, the baseline assessments will be delivered at T=12 weeks and the post intervention assessments will be delivered at T=20 weeks. The primary outcome measure will be delivered at regular intervals of 4 weeks throughout the course of the study (see Table 1).

**Table 1.** Assessment timeline. Informants for each assessment are included in brackets (P – Parent; T – Teacher; YP – Young Person). Assessment acronyms: BAI – Beck’s Anxiety Inventory; IAQ – Intervention Acceptability Questionnaire; PEERS – Program for Education and Enrichment of Relational Skills; PEERS QSQ – PEERS Quality of Socialisation Questionnaire; PEERS TASSK – PEERS Test of Adolescent Social Skills Knowledge; RSE – Rosenberg Self-esteem Scale; SCP – Social Competence with Peers; SDQ – Strengths and Difficulties Questionnaire; SRS – Social Responsiveness Scale; SASI - Schedules for the Assessment of Social Intelligence; SWS – Spence Social Worries Scale; WAIS-Wechsler Adult Intelligence Scale.

Timeline	Assessments
t=0	SCP (P) 1 PEERS Screener (P,YP) SASI (YP) WAIS (YP)
4 weeks	SCP (P) 2
8 weeks	SCP (P) 3
**Baseline** 12 weeks	SCP (P,YP,T) 4 SWS (P,YP,T) PEERS QSQ (P) PEERS TASSK (YP) RSE (YP) BAI (YP) SRS (P,T) SDQ (P,T)
16 weeks	SCP (P) 5
**Post-intervention** 20 weeks	SCP (P) 6 SWS (P,YP,T) PEERS QSQ (P) PEERS TASSK (YP) RSE (YP) BAI (YP) SRS (P,T) SDQ (P,YP,T) IAQ (P,YP) SASI (YP)
24 weeks	SCP (P) 7
28 weeks	SCP (P) 8
**Follow-up** 32 weeks	SCP (P,YP,T) 9

### Screening assessments

*Development and Wellbeing Assessment (DAWBA):* The DAWBA will be used to collect information on the child’s behavioural adjustment and mental health. The DAWBA has been used both in UK national and international surveys ^25– 28^. The DAWBA data will be reviewed by a psychiatrist in accordance with the ICD-10/DSM-V diagnostic criteria. This methodology has been used successfully to gather data of high quality by parental online report. The DAWBA autism module includes a social aptitude scale (SAS) which measures social understanding and social ability (Liddle *et al*., 2009). Participants displaying significant difficulties in the SAS will be eligible for the intervention. The DAWBA is available in 26 languages. The DAWBA will be completed online by parents.

*Strengths and Difficulties Questionnaire (SDQ):* The SDQ is a brief behavioural screening questionnaire ^29^. The SDQ includes scales that measure emotional symptoms, conduct problems, hyperactivity/inattention difficulties, peer relationship problems and prosocial behaviour. The first four scales are combined to create a total difficulties score. An additional impact scale measures the impact of this composite score on daily life. Participants scoring poorly on the peer relationships subscale will be eligible for the intervention. It has been validated for use in children aged 4–17 in UK National studies of psychological adjustment, and a new form for 18+ years old has recently been developed. It will be completed online by the adolescents, parents and teachers.

*Social Responsiveness Scale (SRS):* The SRS measures the severity of autistic traits and the instrument has convergent validity with other ASD diagnostic tools ^30, 31^. The SRS subscales measure Social Awareness, Social Cognition, Social Communication, Social Motivation, and Restricted Interests and Repetitive Behaviour. The SRS will be administered online to parents and teachers.

*Health Questionnaire (HQ):* The questionnaire was developed by the UCLH Turner Syndrome Life Course Project to record information about physical health, health care, education, social life, physical activity and relationships ^32^. The self-report version of the questionnaire will be completed by adolescents. 

*Schedules for the Assessment of Social Intelligence (SASI):* The SASI is a socio-cognitive assessment that measures facial expression recognition, face recognition memory, gaze-monitoring and theory of mind. The SASI is sensitive to subtle deficits in social cognition and has been shown to have excellent reliability and validity ^33^. Adolescents will be asked to complete the SASI online.

*Wechsler Adult Intelligence Scale - Fourth UK Edition (WAIS-IV UK):* The WAIS-IV is an IQ test which measures verbal comprehension, perceptual reasoning, working memory and processing speed. It has been widely used and validated ^34^. It will be administered to adolescents in person.

*PEERS Screener:* The PEERS Screener Questionnaire assesses the participant’s motivation to take part in the PEERS intervention ^18^. It will be administered to parents and adolescents over the phone or in person. Only participants motivated to take part will be considered for the intervention.

### Primary outcome measures

*Social Competence with Peers (SCP):* The SCP assesses the consequences of young people’s interactions with peers, such as the existence and duration of friendships or social invitations ^35^. A modified version of the SCP will be used to adapt the tool for use in young adults. The adolescent group and the parent group will be asked to complete the SCP at regular intervals (every 4 weeks) from baseline to follow-up. Teachers will be asked to complete the SCP at baseline, post-intervention and follow-up.

### Secondary outcome measures

*Strengths and Difficulties Questionnaire (SDQ):* Described previously. It will be completed by the young people, parents and teachers at baseline and post-intervention.

*Social Responsiveness Scale (SRS):* Described previously. It will be completed online by parents and teachers at baseline and post-intervention.

*Spence Social Worries Scale (SWS):* The Spence Social Worries Scale is a psychological questionnaire designed to identify symptoms of social phobia and other forms of anxiety, in children and adolescents. The parent and teacher forms are reported to have excellent internal validity ^35^. It will be completed online by the adolescents, parents and teachers at baseline and post-intervention.

*Schedules for the Assessment of Social Intelligence (SASI):* Described previously. It will be administered online to the adolescent at baseline and post-intervention.

*PEERS Test of Adolescent Social Skills Knowledge (TASSK):* The TASSK is a questionnaire designed to evaluate what the participants have learned from the intervention ^18^. This is the only outcome measure to evaluate changes in social knowledge. It will be administered to the adolescents at baseline and post-intervention.

*PEERS Quality of Socialisation Questionnaire (QSQ):* The QSQ is designed to evaluate the quality of young people’s socialization and frequency of get-togethers ^18^. It will completed online by the parents at baseline and post-intervention.

*Rosenberg Self-esteem Scale (RSE):* The RSE scale is assesses global self-esteem ^36^. It will completed online by the adolescent at baseline and post-intervention.

*Beck’s Anxiety Inventory (BAI):* This scale is a self-report measure used for measuring the severity of anxiety in children and adults ^37^. It will be completed online by the parent and adolescent groups at baseline and post-intervention.

*Camouflaging measure (CAT-Q):* The CAT-Q measures camouflaging (e.g. strategies to mask or compensate autistic characteristics) behaviour in social situations. It is comprised of 25 items and has high internal reliability in autistic adults. Its subscales measure compensation, masking and assimilation ^38^. The CAT-Q will be completed by adolescents.

*Intervention Acceptability Questionnaire (IAQ):* The IAQ has been developed for the study to assess parent and adolescent satisfaction with the intervention ( Supplementary File 1). It will be completed by the parent and adolescent groups once the intervention has ended.

### Missing data and intervention adherence

The occurrence of missing data will be reported for each questionnaire and study time point. Participant intervention adherence, planned absences and study dropouts will be recorded and reported. When possible the causes for missing data, absences or dropout will be reported. Families that miss sessions will be caught up over the phone or conference call before the next session.

### Adverse events

Adverse events will be recorded.

### Statistical analysis

The primary outcome measure (SCP questionnaire) ^16^ will be analysed using visual analysis and multi-level modelling to track individual participant changes over 9 months from baseline to follow up ^39^.

The secondary outcome measures will be analysed for pre-post differences. Data will be analysed using SPSS version 22 statistical software. It is likely that we will be underpowered to detect any significant statistical differences between the pre and post intervention scores; therefore effect sizes (Cohen’s d) will also be calculated. The parent, teacher and adolescent responses to the questionnaires will also be compared to investigate the consistencies between different informants.

We anticipate that adolescent informants will report the greatest positive changes compared to other informants. We also anticipate that the adolescents will report greater improvements on the social knowledge on the TASSK, than on the social performance on the SCP or SDQ (prosocial or peer scale) and social cognition on the SASI. We also expect to see secondary improvements on adolescent self-reports of anxiety on the BAI raw score, social anxiety on the SWS raw total score and self-esteem on the RSE raw total score. We expect to see an increase in camouflaging on the CAT-Q on all the subscales.

In line with previous social skills intervention research we anticipate that positive changes in social performance will be noted by the parents, but that schoolteachers will not observe a change post intervention on the SRS, SDQ and SWS. Specifically we expect parents to report improvements in the SWS total raw score, as well as improvements on the SDQ raw prosocial scale and peer difficulties scale, and improvements on the SRS social communication scale and repetitive and ritualised behaviours scale.

The acceptability of the intervention to families will be assessed using the IAQ. Descriptive statistics will be used to summarise the responses alongside a qualitative summary of the open text answers. We expect that most families will report having positive experiences of the PEERS programme. Based on previous randomised controlled trials we predict that adherence will be on average 80% and that up to two participating families may dropout (Laugeson *et al.,* 2015; Schohl *et al.,* 2014).

## Ethics and dissemination

### Ethics and consent

All participants (young people aged 16–20 and their parent) will give written informed consent prior to entry to the SOAR study. The study has been approved by the West London GTAC Ethics Committee (IRAS: 219817).

### Dissemination

The results of the study will be disseminated at the Turner Syndrome Support Society conference, the study website, at international research conferences and in research articles published in peer-reviewed journals.

## Discussion

This is the first study to pilot a social skills training program with adolescents and young women with TS. Given the PEERS program’s success with teenagers on the spectrum, it is anticipated that young women with TS will also benefit from taking part.

This pilot study has been designed to take an approach of high internal validity. This approach is appropriate given that it is a feasibility pilot conducted with a small number of participants (n=6–10), however the disadvantage of the approach is that the study has low external validity, which reduces the generalizability of the findings. This study will need to be replicated with young people with different social skills profiles, intellectual ability and hormone treatment status.

To our knowledge this will also be the first trial of PEERS delivered online and offline. TS is a rare genetic disorder and the delivery of the full program face to face would have resulted in many families being excluded due to geographical constraints. The program’s acceptability to families will be assessed and this feedback will be used to inform future replications of the intervention. Should the combination of online and offline prove successful, this will enable the to program to be made more widely available.

When assessing social skills it is important to employ a range of assessment tools, which assess different domains of social skills (social knowledge, performance and cognition), as well as a variety of informants ^40, 41^. Meta-analyses of social skills intervention studies show that parents and young people report changes in social skills after taking part in social skills interventions. However, these improvements are rarely reported by teachers ^41, 42^. There is a trend for young people to overestimate the changes in their social skills compared to other informants ^41, 42^. However, a recent meta-analysis of the young person self-report measures suggests that the improvements relate to changes in their social knowledge rather than their social performance ^41^.

The assessment battery has been designed to measure changes in social skills, in the domains of social performance, social knowledge and social cognition. These outcomes will be reported on by the parents, teachers and the young people themselves. Teachers and parents will be asked to report on changes in social performance through questionnaires. The young people will complete questionnaires which measure social performance and social knowledge, as well as an online task to measure changes in social cognition. The maintenance of any potential treatment gains in social performance will be assessed by the parent report at a 3 month follow-up.

It is likely that the adolescent and parent reports will be prone to expectancy biases ^43^. They may exaggerate treatment effects due to their investment in taking part in the intervention. Using external observers (such as teachers or blinded study administrative assessors) is essential to help understand these biases and assess whether changes in performance generalise to other settings ^41, 44^. Unfortunately, due to the small scale of this project, assessments by external observers will not be feasible.

Meta-analyses of social skills interventions for children on the autistic spectrum using the SRS have shown that the largest treatment gains are made in the social communication and repetitive and ritualised behaviours scale ^21^. The changes in repetitive and ritualised behaviours may be mediated by reductions in anxiety or increases in social awareness ^21, 45^. The majority of the participants included in the meta-analyses were adolescent males, therefore it remains to be seen whether these patterns of improvement will be replicated in females with TS.

This study will also use a novel measure of social camouflaging ^46^. Social camouflaging is a strategy adopted by people on the spectrum to manage social situations. It has been likened to wearing a ‘social mask’, where the individual puts on ‘their best self’ ^46^. Camouflaging typically involves masking and compensating for social deficits ^46– 48^. This might involve consciously performing a range of non-verbal cues such as making eye contact during conversations and imitating facial expressions and gestures, or following learnt social scripts such as using pre-prepared jokes or comments ^49^. Recent research suggests that females are better at camouflaging than males ^48, 50^. We anticipate that the intervention will help the participants become more aware of their camouflaging and help them to camouflage more effectively if they choose to use it as a strategy.

## Conclusion

This will be the first social skills training programme trialled with adolescents and young women with TS. Should the trial prove successful, the initial results will be used to inform the sample size for a future randomised controlled trial. Additionally, neither research trials using the PEERS program exclusively in girls, nor trials delivering PEERS online have been published. Therefore, this trial may have a broader impact on the development of treatment strategies for both for young women that experience social skills difficulties (including those on the autistic spectrum), but also for broadening access to treatment by using technology.

## Data availability

No data are associated with the article.

## Supplementary material

Supplementary File 1: Young Person Intervention Acceptability Questionnaire; Parent Intervention Acceptability Questionnaire.
