# Supplementary material for: Protocol: New approaches to managing the social deficits of Turner Syndrome using the PEERS program
Source: F1000Res. 2019 Mar 18;7:1864. Originally published 2018 Nov 29. [Version 2] doi: 10.12688/f1000research.15489.2 (PMC6456833; doi:10.12688/f1000research.15489.2)
Supplement: Supplementary file 1 [file f1000research-7-20091-s0000.tgz › 11c10ccd-58c4-4aae-9a59-faae54f58f0a_Supplementary_File_1_v2.docx]

**Young Person Intervention Acceptability Questionnaire**

*Please tick the appropriate answer*

| 1. How helpful did you find this session? | Not helpful | A little helpful | | Very helpful | |
| --- | --- | --- | --- | --- | --- |
| Introduction and conversational skills I: trading Information | **□** | **□** | | **□** | |
| Conversation skills II: two-way conversations | **□** | **□** | | **□** | |
| Conversation skills III: electronic communication | **□** | **□** | | **□** | |
| Choosing appropriate friends | **□** | **□** | | **□** | |
| Use of humour | **□** | **□** | | **□** | |
| Peer Entry I: entering a conversation | **□** | **□** | | **□** | |
| Peer Entry II: exiting a conversation | **□** | **□** | | **□** | |
| Get-togethers | **□** | **□** | | **□** | |
| Good sportsmanship | **□** | **□** | | **□** | |
| Handling teasing and embarrassing feedback | **□** | **□** | | **□** | |
| Handling bullying and bad reputations | **□** | **□** | | **□** | |
| Handling disagreements | **□** | | **□** | | **□** |
| Rumours and gossip | **□** | | **□** | | **□** |

**2. How would you rate the PEERS group overall?**

**□** Not helpful **□** A little helpful **□** Very helpful

Please let us more about your experience of taking part in the social group:

………………………………………………………………………………………………………………………………………………………………………………………………………………………………………………………………………………………………………………………….

………………………………………………………………………………………………………………………………………………………………………………………………………………………………………………………………………………………………………………………….

………………………………………………………………………………………………………………………………………………………………………………………………………………………………………………………………………………………………………………………….

**3. Do you feel that the PEERS group helped you improve your social skills?**

**□**  No **□**  A little **□**  A lot

Please tell us more:

………………………………………………………………………………………………………………………………………………………………………………………………………………………………………………………………………………………………………………………….

………………………………………………………………………………………………………………………………………………………………………………………………………………………………………………………………………………………………………………………….

………………………………………………………………………………………………………………………………………………………………………………………………………………………………………………………………………………………………………………………….

………………………………………………………………………………………………………………………………………………………………………………………………………………………………………………………………………………………………………………………….

……………………………………………………………………………………………………………………………………………………………

**4. Has taking part in the PEERS programme made you feel more confident in social situations?**

**□**  No **□**  A little **□**  A lot

Please tell us more:

………………………………………………………………………………………………………………………………………………………………………………………………………………………………………………………………………………………………………………………….

………………………………………………………………………………………………………………………………………………………………………………………………………………………………………………………………………………………………………………………….

………………………………………………………………………………………………………………………………………………………………………………………………………………………………………………………………………………………………………………………….

………………………………………………………………………………………………………………………………………………………………………………………………………………………………………………………………………………………………………………………….

……………………………………………………………………………………………………………………………………………………………

**5. Has taking part in the PEERS programme made you feel less anxious about social situations?**

**□**  No **□**  A little **□**  A lot

Please tell us more:

………………………………………………………………………………………………………………………………………………………………………………………………………………………………………………………………………………………………………………………….

………………………………………………………………………………………………………………………………………………………………………………………………………………………………………………………………………………………………………………………….

………………………………………………………………………………………………………………………………………………………………………………………………………………………………………………………………………………………………………………………….

………………………………………………………………………………………………………………………………………………………………………………………………………………………………………………………………………………………………………………………….

……………………………………………………………………………………………………………………………………………………………

**6. Please tell us what you thought of the online meeting rooms:**

………………………………………………………………………………………………………………………………………………………………………………………………………………………………………………………………………………………………………………………….

………………………………………………………………………………………………………………………………………………………………………………………………………………………………………………………………………………………………………………………….

………………………………………………………………………………………………………………………………………………………………………………………………………………………………………………………………………………………………………………………….

………………………………………………………………………………………………………………………………………………………………………………………………………………………………………………………………………………………………………………………….

……………………………………………………………………………………………………………………………………………………………

……………………………………………………………………………………………………………………………………………………………

……………………………………………………………………………………………………………………………………………………………

**7. Please tell us what you thought of the face to face sessions:**

………………………………………………………………………………………………………………………………………………………………………………………………………………………………………………………………………………………………………………………….

………………………………………………………………………………………………………………………………………………………………………………………………………………………………………………………………………………………………………………………….

………………………………………………………………………………………………………………………………………………………………………………………………………………………………………………………………………………………………………………………….

………………………………………………………………………………………………………………………………………………………………………………………………………………………………………………………………………………………………………………………….

……………………………………………………………………………………………………………………………………………………………

……………………………………………………………………………………………………………………………………………………………

……………………………………………………………………………………………………………………………………………………………

**8. What did you learn from the Awkward Social Situations Game?**

………………………………………………………………………………………………………………………………………………………………………………………………………………………………………………………………………………………………………………………….

………………………………………………………………………………………………………………………………………………………………………………………………………………………………………………………………………………………………………………………….

………………………………………………………………………………………………………………………………………………………………………………………………………………………………………………………………………………………………………………………….

………………………………………………………………………………………………………………………………………………………………………………………………………………………………………………………………………………………………………………………….

……………………………………………………………………………………………………………………………………………………………

……………………………………………………………………………………………………………………………………………………………

**9. Did you enjoy taking part in the PEERS programme?**

**□**  No **□**  A little **□**  A lot

Please tell us more:

………………………………………………………………………………………………………………………………………………………………………………………………………………………………………………………………………………………………………………………….

………………………………………………………………………………………………………………………………………………………………………………………………………………………………………………………………………………………………………………………….

………………………………………………………………………………………………………………………………………………………………………………………………………………………………………………………………………………………………………………………….

………………………………………………………………………………………………………………………………………………………………………………………………………………………………………………………………………………………………………………………….

……………………………………………………………………………………………………………………………………………………………

**10. Would you recommend taking part in the PEERS programme to a friend?**

**□**  No **□**  A little **□**  A lot

Please tell us more:

………………………………………………………………………………………………………………………………………………………………………………………………………………………………………………………………………………………………………………………….

………………………………………………………………………………………………………………………………………………………………………………………………………………………………………………………………………………………………………………………….

………………………………………………………………………………………………………………………………………………………………………………………………………………………………………………………………………………………………………………………….

………………………………………………………………………………………………………………………………………………………………………………………………………………………………………………………………………………………………………………………….

……………………………………………………………………………………………………………………………………………………………

**11. Do you feel like you have changed since taking part in the PEERS programme?**

**□**  No **□**  Possibly **□**  Definitely

Please tell us more:

………………………………………………………………………………………………………………………………………………………………………………………………………………………………………………………………………………………………………………………….

………………………………………………………………………………………………………………………………………………………………………………………………………………………………………………………………………………………………………………………….

………………………………………………………………………………………………………………………………………………………………………………………………………………………………………………………………………………………………………………………….

………………………………………………………………………………………………………………………………………………………………………………………………………………………………………………………………………………………………………………………….

……………………………………………………………………………………………………………………………………………………………

**12. Any other comments or feedback:**

………………………………………………………………………………………………………………………………………………………………………………………………………………………………………………………………………………………………………………………….

………………………………………………………………………………………………………………………………………………………………………………………………………………………………………………………………………………………………………………………….

………………………………………………………………………………………………………………………………………………………………………………………………………………………………………………………………………………………………………………………….

………………………………………………………………………………………………………………………………………………………………………………………………………………………………………………………………………………………………………………………….

……………………………………………………………………………………………………………………………………………………………

**Parent Intervention Acceptability Questionnaire**

*Please tick the appropriate answer*

| 1. How helpful did you find this session? | Not helpful | A little helpful | | Very helpful | |
| --- | --- | --- | --- | --- | --- |
| Introduction and conversational skills I: trading Information | **□** | **□** | | **□** | |
| Conversation skills II: two-way conversations | **□** | **□** | | **□** | |
| Conversation skills III: electronic communication | **□** | **□** | | **□** | |
| Choosing appropriate friends | **□** | **□** | | **□** | |
| Use of humour | **□** | **□** | | **□** | |
| Peer Entry I: entering a conversation | **□** | **□** | | **□** | |
| Peer Entry II: exiting a conversation | **□** | **□** | | **□** | |
| Get-togethers | **□** | **□** | | **□** | |
| Good sportsmanship | **□** | **□** | | **□** | |
| Handling teasing and embarrassing feedback | **□** | **□** | | **□** | |
| Handling bullying and bad reputations | **□** | **□** | | **□** | |
| Handling disagreements | **□** | | **□** | | **□** |
| Rumours and gossip | **□** | | **□** | | **□** |
| Where do go from here | **□** | | **□** | | **□** |

**2. How would you compare your daughter’s social ability prior to PEERS with her social ability now:**

**□** Worse **□** The same **□** Improved

Please tell us more:

………………………………………………………………………………………………………………………………………………………………………………………………………………………………………………………………………………………………………………………….………………………………………………………………………………………………………………………………………………………………………………………………………………………………………………………………………………………………………………………….………………………………………………………………………………………………………………………………………………………………………………………………………………………………………………………………………………………………………………………….………………………………………………………………………………………………………………………………………………………………………………………………………………………………………………………………………………………………………………………….……………………………………

**3. For your daughter, this intervention was:**

**□** Not helpful **□** A little helpful **□** Very helpful

Please let us know what you found helpful or unhelpful:

………………………………………………………………………………………………………………………………………………………………………………………………………………………………………………………………………………………………………………………….………………………………………………………………………………………………………………………………………………………………………………………………………………………………………………………………………………………………………………………….………………………………………………………………………………………………………………………………………………………………………………………………………………………………………………………………………………………………………………………….………………………………………………………………………………………………………………………………………………………………………………………………………………………………………………………………………………………………………………………….……………………………………

**4. The parent training group was:**

**□** Not helpful **□** A little helpful **□** Very helpful

Please let us know what you found helpful or unhelpful:

………………………………………………………………………………………………………………………………………………………………………………………………………………………………………………………………………………………………………………………….………………………………………………………………………………………………………………………………………………………………………………………………………………………………………………………………………………………………………………………….………………………………………………………………………………………………………………………………………………………………………………………………………………………………………………………………………………………………………………………….………………………………………………………………………………………………………………………………………………………………………………………………………………………………………………………………………………………………………………………….

…………………………………………………………………………………………………………………………………………………………….

**5. Overall, how would you rate the PEERS group that your daughter attended?**

**□** Not helpful **□** A little helpful **□** Very helpful

Please tell us more:

………………………………………………………………………………………………………………………………………………………………………………………………………………………………………………………………………………………………………………………….………………………………………………………………………………………………………………………………………………………………………………………………………………………………………………………………………………………………………………………….………………………………………………………………………………………………………………………………………………………………………………………………………………………………………………………………………………………………………………………….………………………………………………………………………………………………………………………………………………………………………………………………………………………………………………………………………………………………………………………….

…………………………………………………………………………………………………………………………………………………………….

**6. Please tell us what you thought of the online meeting rooms:**

………………………………………………………………………………………………………………………………………………………………………………………………………………………………………………………………………………………………………………………….

………………………………………………………………………………………………………………………………………………………………………………………………………………………………………………………………………………………………………………………….

………………………………………………………………………………………………………………………………………………………………………………………………………………………………………………………………………………………………………………………….

………………………………………………………………………………………………………………………………………………………………………………………………………………………………………………………………………………………………………………………….

……………………………………………………………………………………………………………………………………………………………

……………………………………………………………………………………………………………………………………………………………

……………………………………………………………………………………………………………………………………………………………

**7. Please tell us what you thought of the face to face sessions:**

………………………………………………………………………………………………………………………………………………………………………………………………………………………………………………………………………………………………………………………….

………………………………………………………………………………………………………………………………………………………………………………………………………………………………………………………………………………………………………………………….

………………………………………………………………………………………………………………………………………………………………………………………………………………………………………………………………………………………………………………………….

………………………………………………………………………………………………………………………………………………………………………………………………………………………………………………………………………………………………………………………….

**8. Is there anything we could have done differently?**

………………………………………………………………………………………………………………………………………………………………………………………………………………………………………………………………………………………………………………………….………………………………………………………………………………………………………………………………………………………………………………………………………………………………………………………………………………………………………………………….………………………………………………………………………………………………………………………………………………………………………………………………………………………………………………………………………………………………………………………….………………………………………………………………………………………………………………………………………………………………………………………………………………………………………………………………………………………………………………………….

………………………………………………………………………………………………………………………………………………………………………………………………………………………………………………………………………………………………………………………….

**9. Would you recommend taking part in the PEERS programme to other families?**

**□**  No **□**  Possibly **□**  Definitely

Please tell us more:

………………………………………………………………………………………………………………………………………………………………………………………………………………………………………………………………………………………………………………………….

………………………………………………………………………………………………………………………………………………………………………………………………………………………………………………………………………………………………………………………….

………………………………………………………………………………………………………………………………………………………………………………………………………………………………………………………………………………………………………………………….

………………………………………………………………………………………………………………………………………………………………………………………………………………………………………………………………………………………………………………………….

……………………………………………………………………………………………………………………………………………………………

………………………………………………………………………………………………………………………………………………………………………………………………………………………………………………………………………………………………………………………….

……………………………………………………………………………………………………………………………………………………………
