# Supplementary material for: Protocol: New approaches to managing the social deficits of Turner Syndrome using the PEERS program
Source: F1000Res. 2019 Mar 18;7:1864. Originally published 2018 Nov 29. [Version 2] doi: 10.12688/f1000research.15489.2 (PMC6456833; doi:10.12688/f1000research.15489.2)
Supplement: Peer review history

# Peer review: Reviewer response for version 2

**DOI:** 10.5256/f1000research.20091.r45871

**Published:** 2019-04-05

**Authors:** David E. Sandberg

**Affiliations:** 1 Department of Pediatrics, Medical School, University of Michigan, Ann Arbor, MI, USA

I thank the authors for their responses. I have no further comments.

I have read this submission. I believe that I have an appropriate level of expertise to confirm that it is of an acceptable scientific standard.

# Peer review: Reviewer response for version 2

**DOI:** 10.5256/f1000research.20091.r45872

**Published:** 2019-03-19

**Authors:** Claus H. Gravholt

**Affiliations:** 1 Department of Endocrinology and Internal Medicine, Aarhus University Hospital, Aarhus, Denmark

I have no further comments. The authors have dealt with all questions raised.

I have read this submission. I believe that I have an appropriate level of expertise to confirm that it is of an acceptable scientific standard.

# Peer review: Reviewer response for version 1

**DOI:** 10.5256/f1000research.16884.r41839

**Published:** 2019-01-15

**Authors:** Claus H. Gravholt

**Affiliations:** 1 Department of Endocrinology and Internal Medicine, Aarhus University Hospital, Aarhus, Denmark

Wolstencroft et al presents a pilot protocol designed to improve the social cognition of adolescents with Turner syndrome (TS) based on the PEERS program. Overall, this study is very interesting and timely, given that many with TS have a social skills deficit.

 I have some comments:

1. Design and sample size: uncontrolled, with the aim of including 6-10 TS. The uncontrolled design is acceptable in a pilot trial. I’m more worried about the rather low n. There is a large variability, perhaps even larger than among normal females, in the presentation of females with TS and that may not be captured satisfactorily with a n of 6-10. However, one could ask if it is at all necessary to perform a pilot study, given that this program has shown to be a success in other study groups?
2. Inclusion criteria: the inclusion criteria are rather strict, and I think that the authors will end up excluding a rather large proportion of females with TS, which is a pity. Many females that in their youth may not present with social skills difficulties, will actually do this at a later age, and I think it would be interesting to have some of these females included as well. Can females with hearing difficulties, but treated with a hearing aid, be included?
3. The intervention program, PEERS, is certainly very relevant.
4. The scales used to monitor effect seem relevant. The primary and secondary outcomes are relevantly described.
5. The intervention program seems rather massive with multiple scales and 12 times 90 minutes interventions. Have the authors considered how this will affect the participation rate in the study? I guess they must have contemplated this. Are there experience from other groups of patients? The authors expect 2 family dropouts – and if the inclusion ends at 6 families, that would then leave 4 families – hardly enough to call it a pilot study?
6. Conclusively, if this pilot study proves successful, it will be a welcome addition to the program of care established by excellence center for TS around the world.

I have read this submission. I believe that I have an appropriate level of expertise to confirm that it is of an acceptable scientific standard, however I have significant reservations, as outlined above.

# Response

**Published:** 2019-02-20

**Authors:** Jeanne Wolstencroft

**Affiliations:** UCL Great Ormond Street Institute of Child Health, UK

Dear Claus Gravholt,

 Thank you for your comments. We are encouraged to hear that you are convinced of the value of using the PEERS protocol with young women with TS. We have addressed your concerns in turn below:

 1.As you correctly point out, there is large variability between young women with TS. Our pilot will only recruit young women experiencing difficulties with friendships who wish to improve their social skills, but within this group there will still be a substantial amount of variability. We are currently conducting a survey of mental health and social skills difficulties in TS (SOAR Study). Our preliminary findings indicate that girls and women with TS have significantly more peer interaction problems when compared to population female norms as measured by the Strengths and Difficulties Questionnaire (Goodman et al., 2010). On our measure of autistic symptomatology (SRS-2; Constantino et al., 2012) 40% of young women with TS scored in the normal range, 14.5% in the mild range, 17.1% in the moderate range and 27.4% in the severely impaired range (SOAR study unpublished findings; n=117). Our sample for the PEERS pilot is representative of this range of social skills difficulties; among our enrolled participants, scores lie in the normal to abnormal range on the Strengths and Difficulties subscale for quality of peer-interactions and they also range from normal to severely impaired on the SRS-2.

 We believe it is necessary to conduct a pilot of the PEERS program with young women with TS before conducting a full-scale trial because the program was initially developed to treat adolescent boys with autism. The emerging literature on young women with autism shows clearly that women with social communication impairments face different challenges to those experienced by young men with autistic traits. We will therefore need to adapt the content of the program. For example, some of the lessons focus on issues such as ‘good sportsmanship’ and ‘appropriate uses of humour’; females with TS would not regard these skills as being of core relevance to their social adaptation. Additionally, there is no precedent for delivering social skills training online, therefore there is a need to pilot the acceptability of virtual meeting rooms and to adapt the behavioural rehearsal components of the training to an online environment.

 2.You are correct in noting that many girls with TS have impaired adaptation to the social environment that manifests most obviously once they enter adolescence. We have found that social difficulties emerge and intensify over that period, from the time of entry into secondary education to early adulthood. Therefore, our pilot study’s age range (16-20 years) is designed to help young women at a time when their social difficulties are emerging, and they are becoming aware of them.

 We are not excluding anyone on the grounds of impaired hearing, if that problem is being successfully managed. Two young women who wear hearing aids are currently enrolled in the pilot study. Unfortunately, the program would need to be substantially modified in order to accommodate those with more profound hearing impairments. The inclusion criteria have been clarified in the protocol v2.

 5. As originally devised, the PEERS program required there to be no more than 8-10 participants in the treatment group. The program is very intensive and is characterized by a focus on individual needs as well as on group dynamics. Several staff are required on site to manage the child/parent groups. By adapting the program to be delivered from an online platform we aim to increase its acceptability to participants and to reduce the associated costs. Randomized control trials of PEERS have reported attrition rates of 7-13% (Schohl et al.,2014; Laugeson et al., 2015), hence our prediction that 1-2 participants out of 10 may drop out. However, we are now three-quarters of the way through the pilot trial and we have not yet had any dropouts.

 Best wishes,

 Jeanne Wolstencroft

 References:

 Constantino, J. N., & Gruber, C. P. (2012). Social responsiveness scale–second edition (SRS-2). Torrance: Western Psychological Services.

 Goodman, A., Lamping, D. L., & Ploubidis, G. B. (2010). When to use broader internalising and externalising subscales instead of the hypothesised five subscales on the Strengths and Difficulties Questionnaire (SDQ): data from British parents, teachers and children. Journal of abnormal child psychology, 38(8), 1179-1191.

 Laugeson, E. A., Gantman, A., Kapp, S. K., Orenski, K., & Ellingsen, R. (2015). A randomized controlled trial to improve social skills in young adults with autism spectrum disorder: The UCLA PEERS® program. Journal of autism and developmental disorders, 45(12), 3978-3989.

 Schohl, K. A., Van Hecke, A. V., Carson, A. M., Dolan, B., Karst, J., & Stevens, S. (2014). A replication and extension of the PEERS intervention: examining effects on social skills and social anxiety in adolescents with autism spectrum disorders. Journal of Autism and Developmental Disorders, 44(3), 532-545.

# Peer review: Reviewer response for version 1

**DOI:** 10.5256/f1000research.16884.r41256

**Published:** 2018-12-27

**Authors:** David E. Sandberg

**Affiliations:** 1 Department of Pediatrics, Medical School, University of Michigan, Ann Arbor, MI, USA

The study protocol describes a pilot project that examines the effectiveness of a social skills training program – originally developed for youth on the autism syndrome spectrum – applied to the social skills deficits of adolescent and young women with Turner syndrome (TS)(45,X). The neurocognitive profile of girls and women with TS has been extremely well documented and has repeatedly been shown to be associated with deficits in social cognition and skills. This aspect of the TS phenotype is likely a significant factor accounting for the gap between educational attainment in this population (shown to exceed to population norms), and their occupational status and measures of independence from family caregivers. Women with TS have also been shown to exhibit both delays and arrest in psychosexual milestones which are more than likely linked to the characteristic social behaviour phenotype associated with this karyotype ^1^.

 The investigators should be commended for proposing to adopt a proven efficacious and effective intervention for social skills deficits to potentially modify the behavioural phenotype in TS. The PEERS program is well-suited to the task because of the similarities in social skills deficits in high-functioning ASD and TS. Work on this topic is long overdue.

 The rationale for the pilot study is well described, although the authors have possibly overstated, in the Introduction, the lack of “systematic evaluations of the mental health of young women with TS…”. In fact, there are multiple studies assessing both the psychiatric status and psychosocial/sexual adaptation of this population. What has been sorely missing are psychosocial interventions to potentially ameliorate deficits, and the proposed study is directed precisely toward this objective.

 There are the following elements I found missing from the protocol or require further consideration:

1. p.2 Study Design - it’s unclear what the following refers to: “All participants will be matched for age, degree of social impairment, intellectual ability and hormone therapy treatment.” Each participant will serve as their own control, so I don’t understand the “matching” piece.
2. p. 2. Participant inclusion and exclusion criteria – details are not provided regarding the SOAR questionnaire screening for eligibility based on social skills deficits.
3. Will recruitment be restricted to girls/women with a 45,X karyotype or will those with a variant, including chromosomal mosaicism, be eligible?
4. The Discussion notes that self and parent reports are prone to bias because of expectations regarding the intervention and note that a remedy to overestimating the benefits can come from employing external observers. The investigators justify not employing external observers because of the small scale of this project. However, one could turn that argument around by questioning whether it would be worthwhile to pursue a full-scale trial of PEERS in TS if the effects observed in the pilot are driven by biased reports.

I have read this submission. I believe that I have an appropriate level of expertise to confirm that it is of an acceptable scientific standard, however I have significant reservations, as outlined above.

## References

[1] GravholtCHAndersenNHConwayGSDekkersOMGeffnerMEKleinKOLinAEMaurasNQuigleyCARubinKSandbergDESasTCJSilberbachMSöderström-AnttilaVStochholmKvan Alfen-van derVeldenJAWoelfleJBackeljauwPF: Clinical practice guidelines for the care of girls and women with Turner syndrome: proceedings from the 2016 Cincinnati International Turner Syndrome Meeting. *Eur J Endocrinol*.177(3) : 10.1530/EJE-17-0430 G1-G70 10.1530/EJE-17-0430 28705803

# Response

**Published:** 2019-02-20

**Authors:** Jeanne Wolstencroft

**Affiliations:** UCL Great Ormond Street Institute of Child Health, UK

Dear David Sandberg,

 Thank you for your comments. We are encouraged to hear that you are convinced of the value of using the PEERS protocol with young women with TS. We have addressed your concerns in turn below:

 1.Participants in the treatment group were chosen because they have similar degrees of social impairment and intellectual ability. We have clarified the wording around the ‘matching of participants’ in the protocol v2. You are correct, they will act as their own controls.

 2.Motivation to take part in the intervention is assessed using the PEERS screener interview (Laugeson et al., 2009). It was essential to ensure that the young people and their parents were motivated to take part in the social group in order to minimize potential attrition over the 12 week intervention period. Their social deficits were assessed using a combination of clinical judgement and screening questionnaires:

- Social Aptitude Scale (SAS): The SAS is presented as part of the Development and Wellbeing Assessment’s autism module. The SAS is a ten item parent-report measure which assesses social understanding and social ability (as opposed to peer interaction deficits) (Liddle et al., 2008).
- Strengths and Difficulties Questionnaire (SDQ): The SDQ is a brief behavioural screening questionnaire (Goodman et al., 2010). The SDQ includes scales that measure emotional symptoms, conduct problems, hyperactivity/inattention difficulties, peer relationship problems and prosocial behaviour.

3.Recruitment will not be restricted to young women with a monosomic non-mosaic 45,X karyotype. This has now been clarified in the protocol v2.

 4.Objectively evaluating the outcome of social skills interventions presents a number of challenges. We agree that expectancy biases are likely to occur. Some outcome measures focus only on self-assessment of social performance or social knowledge. Questionnaires that have been designed for parents, teacher or adult observer respondents, may lack ecological validity. To our knowledge, the outcome of PEERS has not yet been measured by peer-ratings of change. We are currently testing a novel methodology that could address this deficiency.

 Currently, we do have some potentially objective measures of change. We obtain teacher ratings of social behaviour, parent reports of changes in the TS girl’s social relationships, and individual increases in social knowledge.

 As we have indicated, the intervention is designed to take account of individual differences. Accordingly, outcomes are diverse. There will be variability within the sample, and it is unlikely that treatment benefits will be captured by standardized questionnaires alone.

 Jeanne Wolstencroft

 References:

 Goodman, A., Lamping, D. L., & Ploubidis, G. B. (2010). When to use broader internalising and externalising subscales instead of the hypothesised five subscales on the Strengths and Difficulties Questionnaire (SDQ): data from British parents, teachers and children. Journal of abnormal child psychology, 38(8), 1179-1191.

 Laugeson, E. A., Frankel, F., Mogil, C., & Dillon, A. R. (2009). Parent-assisted social skills training to improve friendships in teens with autism spectrum disorders. *Journal of autism and developmental disorders*, *39*(4), 596-606.

 Liddle, E. B., Batty, M. J., & Goodman, R. (2009). The social aptitudes scale: an initial validation. *Social psychiatry and psychiatric epidemiology*, *44*(6), 508.
